# Supplementary material for: IL-12p35 induces expansion of IL-10 and IL-35-expressing regulatory B cells and ameliorates autoimmune disease
Source: Nat Commun. 2017 Sep 28;8:719. doi: 10.1038/s41467-017-00838-4 (PMC5620058; doi:10.1038/s41467-017-00838-4)
Supplement: Supplementary file 1 — Supplementary Information [file 41467_2017_838_MOESM1_ESM.pdf]

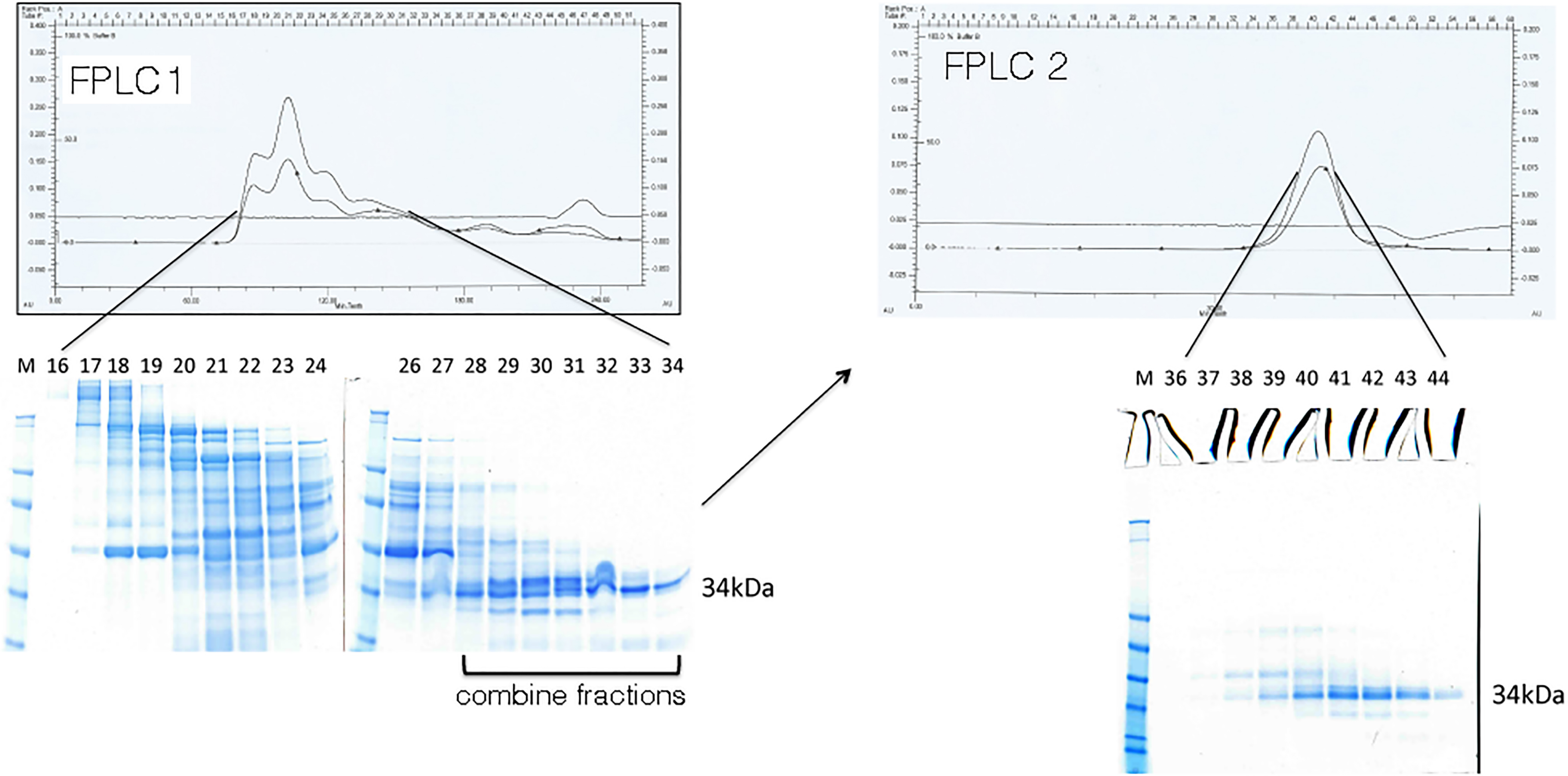

Supplementary Figure 1. Purification and characterization of recombinant mouse Ebi3. High-Five insect cells expressing high levels of the pMTB vector encoding the Ebi3 cDNA were identified by drug (Blasticidin S at 100µg/ml) selection and absence of mutation in the Ebi3 cDNA construct was verified by DNA sequencing. The His-tagged recombinant Ebi3 protein (rEbi3) was sequentially purified usingNi-NTA Purification system, size-exclusion Centricon filtration units and by fractionation on Sephacryl S-200 and Superose-6 FPLC columns. Analysis of the FPLC column fractions was by non-denaturing SDS PAGE and the gels were stained with Coomassie-Blue.

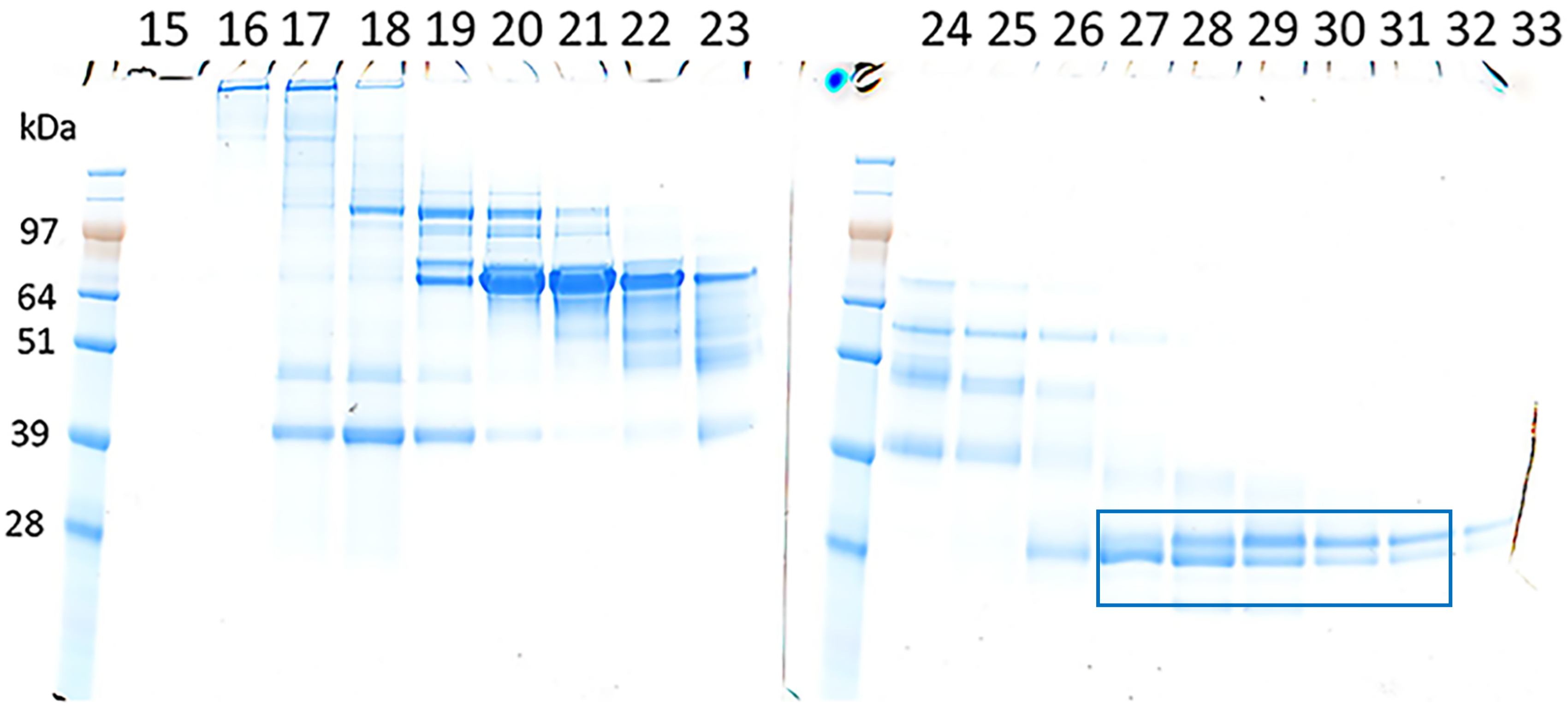

Supplementary Figure 2a. Full scans of Coomassie-Blue gels shown in Figure1c of the manuscript. Cropped areas are marked in blue rectangles.

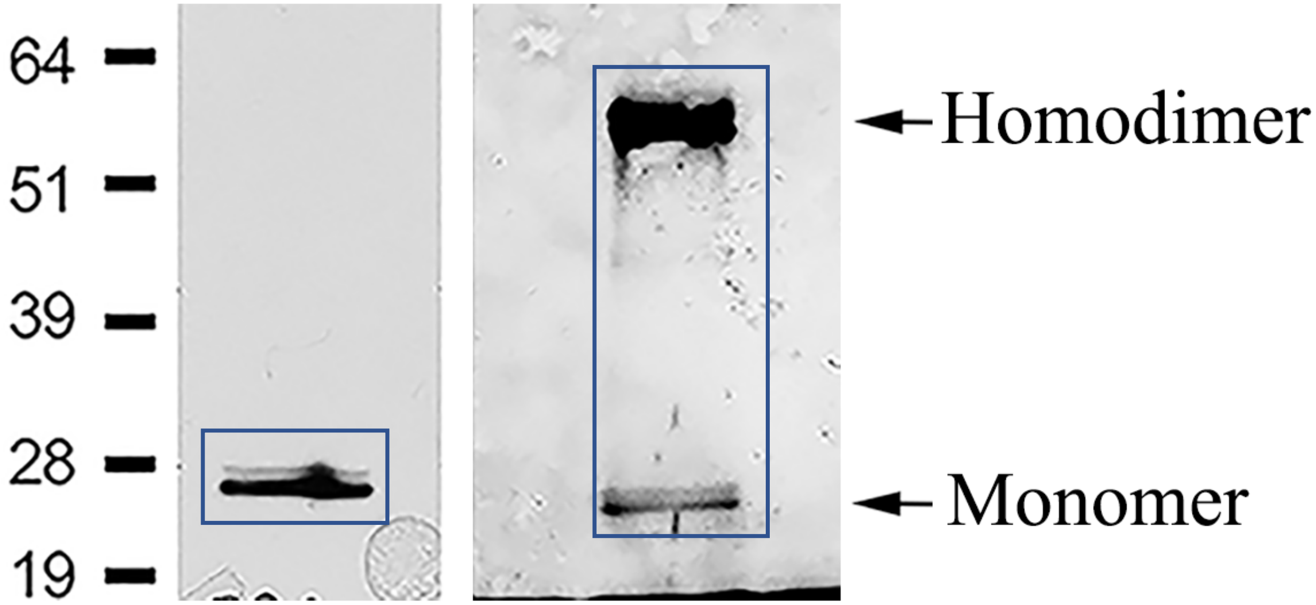

Supplementary Figure 2b. Full scans of gels shown in Figure1b of the manuscript. Cropped areas are marked in blue rectangles.

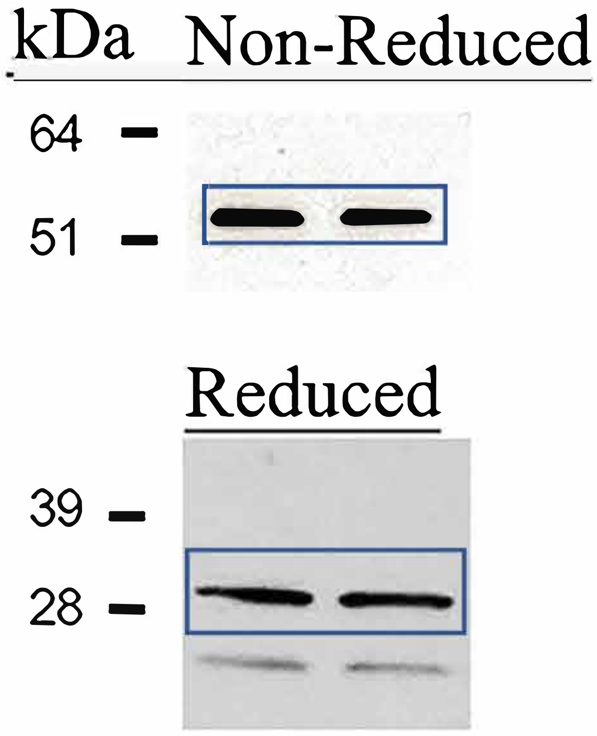

Supplementary Figure 2c. Full scans of gels shown in Figure1c of the manuscript. Cropped areas are marked in blue rectangles.

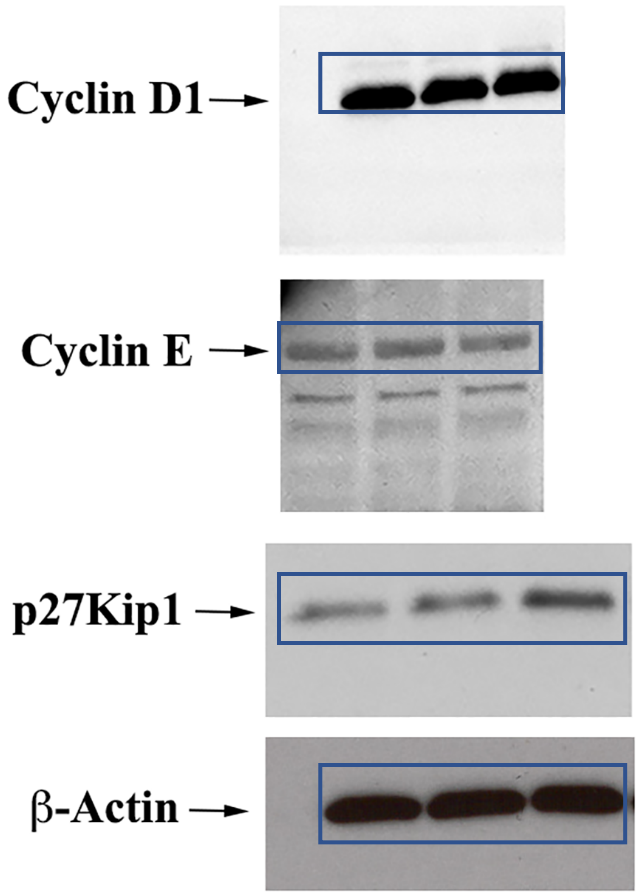

Supplementary Figure 2h. Full scans of gels shown in Figure1h of the manuscript. Cropped areas are marked in blue rectangles.

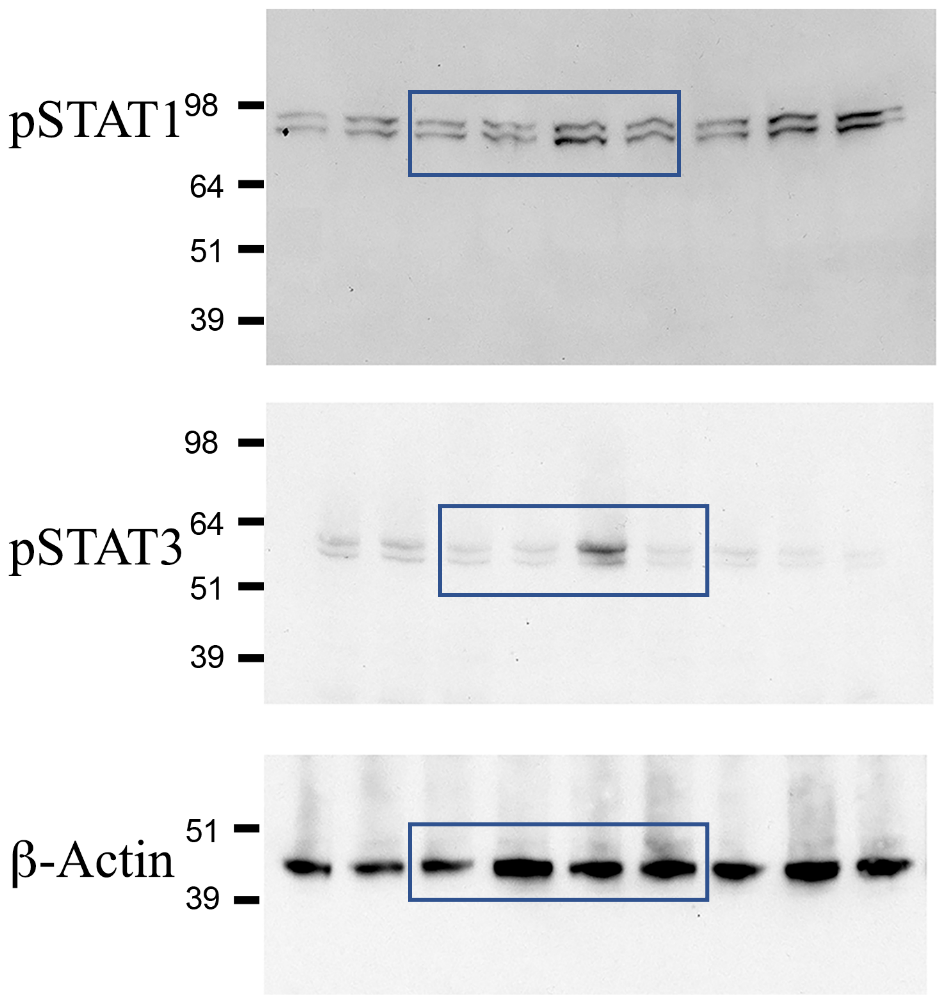

Supplementary Figure 2i. Full scans of gels shown in Figure1i of the manuscript. Cropped areas are marked in blue rectangles.

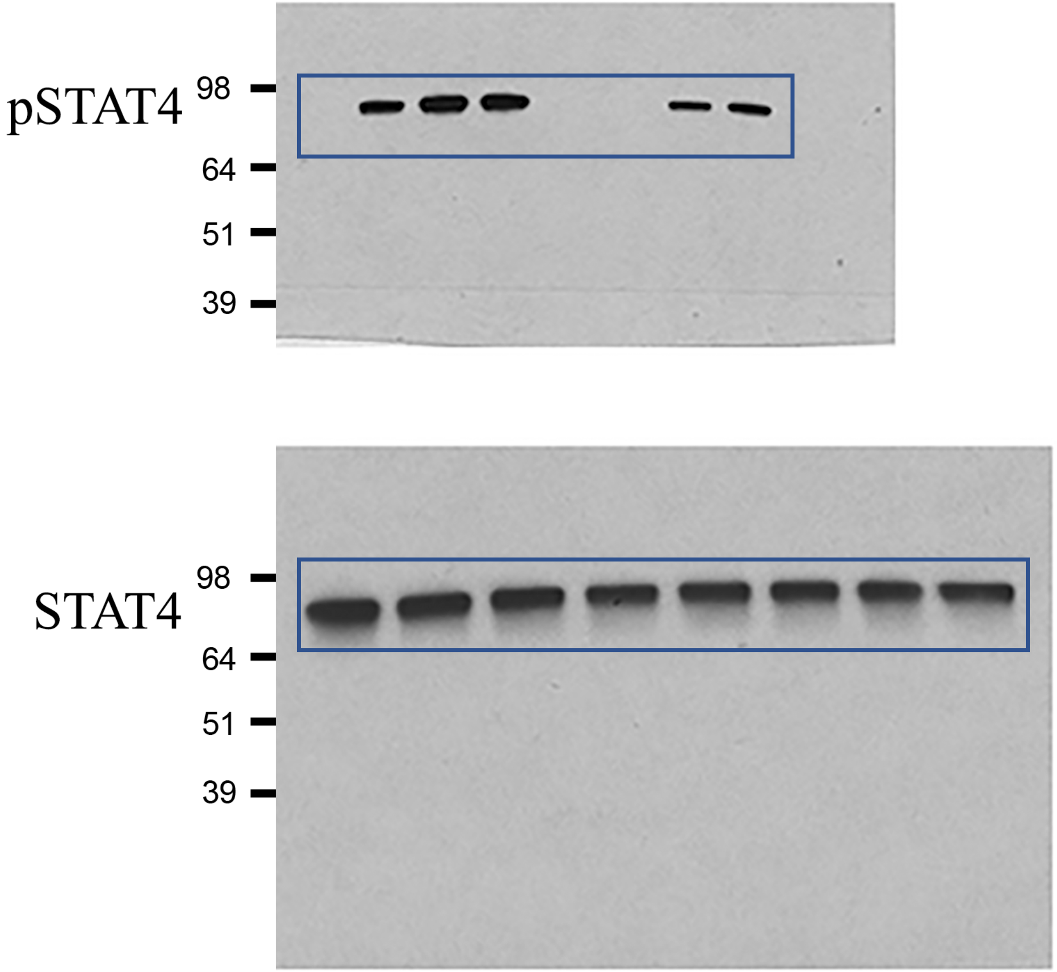

Supplementary Figure 2j. Full scans of gels shown in Figure1j of the manuscript. Cropped areas are marked in blue rectangles.

Table S1. List of primers for qPCR experiments

Species	Gene	Assay ID	Assay Design
Mouse	*IL-12A p35*	Mm00434169_m1	Probe span exons
	EBI3	Mm00469264_g1	Probe span exons
	IL-10	Mm00439616_m1	Probe span exons
	GADPH	Mm99999915_g1	Probe span exons
	β-actin	Mm00469264_g1	Probe span exons

Table S2. List of antibodies for Western blot

Antibody	Manufacture	Catalogy number	Lot number
IL-12A p35 (m-19)	Santa Cruz Biotechnology	SC-9350	B2516
Cyclin E (M-20)	Santa Cruz Biotechnology	SC-481	A028
Cyclin D1 (R-124)	Santa Cruz Biotechnology	SC-6281	L127
p27 Kip1	Cell Signaling	2552	5
Actin (I-19)	Santa Cruz Biotechnology	SC-1616	F3016
pSTAT1 (Y701)	Cell Signaling	9167	15
pSTAT3 (Y705)	Cell Signaling	9131	18
pSTAT4 (Y693)	Cell Signaling	5267	1
STAT4 (C4b10)	Cell Signaling	2653S	3
EBI3 (G-4)	Santa Cruz Biotechnology	SC-166158	H2916
